# Salvage Debridement, Antibiotics and Implant Retention (“DAIR”) With Local Injection of a Selected Cocktail of Bacteriophages: Is It an Option for an Elderly Patient With Relapsing Staphylococcus *aureus* Prosthetic-Joint Infection?

**DOI:** 10.1093/ofid/ofy269

**Published:** 2018-10-24

**Authors:** Tristan Ferry, Gilles Leboucher, Cindy Fevre, Yannick Herry, Anne Conrad, Jérôme Josse, Cécile Batailler, Christian Chidiac, Mathieu Medina, S Lustig, Frédéric Laurent, Lyon BJI Study Group, Tristan Ferry, Tristan Ferry, Florent Valour, Thomas Perpoint, André Boibieux, François Biron, Patrick Miailhes, Florence Ader, Agathe Becker, Sandrine Roux, Claire Triffault-Fillit, Anne Conrad, Alexie Bosch, Fatiha Daoud, Johanna Lippman, Evelyne Braun, Christian Chidiac, Sébastien Lustig, Elvire Servien, Romain Gaillard, Antoine Schneider, Stanislas Gunst, Cécile Batailler, Michel-Henry Fessy, Jean-Luc Besse, Yannick Herry, Anthony Viste, Philippe Chaudier, Cyril Courtin, Lucie Louboutin, Sébastien Martres, Franck Trouillet, Cédric Barrey, Emmanuel Jouanneau, Timothée Jacquesson, Ali Mojallal, Fabien Boucher, Hristo Shipkov, Joseph Chateau, Frédéric Aubrun, Mikhail Dziadzko, Caroline Macabéo, Frederic Laurent, Laeticia Beraud, Jérôme Josse, Camille Kolenda, Céline Dupieux, Fabien Craighero, Loic Boussel, Jean-Baptiste Pialat, Isabelle Morelec, Marc Janier, Francesco Giammarile, Michel Tod, Marie-Claude Gagnieu, Sylvain Goutelle, Béatrice Grisi, Cédric Dananche, Eugénie Mabrut

**Affiliations:** 1 Service de Maladies Infectieuses, Hôpital de la Croix-Rousse, Hospices Civils de Lyon, France; 2 Université Claude Bernard Lyon 1, France; 3 Centre International de Recherche en Infectiologie, Lyon, France; 4 Centre Interrégional de Référence des Infections Ostéo-Articulaires Complexes (CRIOAc Lyon), Hospices Civils de Lyon, France; 5 Pharmacie, Hôpital de la Croix-Rousse, Hospices Civils de Lyon, France; 6 Pherecydes Pharma, Romainville, France; 7 Service de Chirurgie Orthopédique, Hôpital de la Croix-Rousse, Hospices Civils de Lyon, France; 8 Laboratoire de Bactériologie, Institut des Agents Infectieux, Hôpital de la Croix-Rousse, Hospices Civils de Lyon, France

**Keywords:** bacteriophage, DAIR, prosthetic-joint infection, S. aureus, suppressive therapy

## Abstract

Local injection of a bacteriophages mix during debridement, antibiotics and implant retention (“DAIR”) was performed to treat a relapsing Staphylococcus *aureus* chronic prosthetic joint infection (PJI). This salvage treatment was safe and associated with a clinical success. Scientific evaluation of the potential clinical benefit of bacteriophages as antibiofilm treatment in PJI is now feasible and required.

An 80-year-old obese (100 kg) woman with type 2 diabetes mellitus and mild chronic kidney injury (creatinine clearance 60 mL/minute) had history of relapsing prosthetic joint infection (PJI) of the right hip. In brief, the patient has had acute methicillin-susceptible *Staphylococcus aureus* (MSSA) postoperative infection in 2012 treated with debridement, antibiotics and implant retention (DAIR), followed by 1-stage exchange in 2012, then by a 2-stage exchange in 2015 with reimplantation of a large resection prosthetic joint (Figure 1A). A new DAIR was performed for fluoroquinolone-resistant *Escherichiacoli* hematogenous PJI in 2016, and a subsequent DAIR 3 weeks later was required, due to a clinical relapse with persistence of *E coli* in peroperative samples. Suppressive antimicrobial therapy with ceftriaxone (2 g/day) was started. Under therapy, facing suspicion of clinical signs of relapse, the antibiotic was stopped. A purulent discharge appeared (Figure 1B) with a painful hip, elevation of C-reactive protein (156 mg/L), without prosthesis loosening on x-ray. Multidrug-resistant Pseudomonas *aeruginosa* and MSSA (fully susceptible except for penicillin) grew in culture from the swab of the pus. Pherecydes Pharma prepared 3 bacteriophages active against the retrieved *P aeruginosa* strain (based on the results of the phagogram described below). The strain of MSSA was unfortunately not retained, but 3 bacteriophages against *S aureus* were selected from the Pherecydes library according to their broad and complementary spectrum. These bacteriophages, which are still in a development process, are not approved drugs at this time. Although the manufacturers followed the same processes as those established by the Good Manufacturing Practice (GMP) guidelines, they were produced in a research and development (R&D) laboratory (not GMP). The French National Agency for Medicines and Health Products Safety (ANSM) carefully reviewed the quality control tests applied to these batches, in collaboration with the hospital pharmacist and before salvage therapy. Six vials containing 1 mL of 10^10^ plaque-forming units (PFU)/mL suspension of each bacteriophage in Dulbecco’s phosphate-buffered saline were sent to our hospital pharmacist, who mixed the *P aeruginosa* and the *S aureus* phages in 2 different saline solutions of 10 mL as “compounded” drug products (also called “magistral” preparations in Europe). The DAIR procedure (Figure 1C) revealed pus in contact with the prosthesis. Changing the mobile parts of the prosthesis (material not available) was unfortunately not possible. Just before joint closure, both bacteriophage mixes were successively injected into the joint (Figure 1D; video in Supplementary Files). Operative samples confirmed MSSA in culture but not *P aeruginosa*. *Enterococcus faecalis* (susceptible to amoxicillin) and *Staphylococcus lugdunensis* (susceptible to all antibiotics, including penicillin) were also detected (numerous colonies). In addition, the patient was treated with 850 mg/day daptomycin until month 3 and then exclusive oral treatment (6 g/day amoxicillin and 1800 mg/day clindamycin) until month 6. Thereafter, only amoxicillin was continued as suppressive antimicrobial therapy targeting *E faecalis* and *S lugdunensis*. During the follow-up, a new DAIR procedure was performed for a hematogenous *Citrobacter koseri* acute hip infection (*S aureus* did not grow from operative cultures). Ciprofloxacin was added and stopped 2 months later. Eighteen months after the bacteriophage injection (10 months after the *C koseri* infection), still under amoxicillin, the outcome was favorable without any clinical signs of persistent infection (Figure 1E). “Phagogram”, ie, activity of the selected bacteriophages on the *S aureus* strain that grew preoperatively, was done retrospectively. Efficiency of each bacteriophage was tested using efficiency of plating (EOP) and killing assays (Figure 1F). The EOP assay is based on the visualization of bacterial lysis when the strain is spotted on a solid medium (spot test). In case of bacterial lysis with PFU, an EOP score defined by the patient-strain/reference-strain bacteriophage titer is indicated. The closer the score is to 1, the more effective the bacteriophage is. For the killing assay, the patient’s strains were cultured in a 96-well plate at a starting concentration of 1 × 10^6^ colony-forming units/mL with or without bacteriophage. Each bacteriophage was added individually at 3 different concentrations, leading to different multiplicities of infection ([MOIs] ratio of phage/bacteria). The volume of phages added to bacterial cells were calculated to deliver 1, 10, and 100 phages per bacteria. However, under real experimental conditions, the MOIs were different and determined after each phagogram. As a consequence, we refer to them as low, medium, and high MOI. The bacterial concentrations were monitored over time by optical density at 600 nm. Five clones of the patient’s strain were tested with the anti-*S aureus* bacteriophages. Among the bacteriophages used, the 1493 and 1815 showed a clear lytic activity (with visualization of PFU) with high EOP scores (4.4 × 10^−1^ and 7.4 × 10^−1^, respectively). Bacteriophage 1957 was also active, with PFU visualization, but it was less effective (low EOP score: 4.9 × 10^−3^) and displayed no activity on *S aureus* in the killing assay, in comparison with the 2 other bacteriophages. We concluded that bacteriophages 1493 and 1815 were active and effective against this *S aureus* strain, but not phage 1957. In addition, these bacteriophages had no activity against *S lugdunensis* (data not shown).

**Figure 1.**
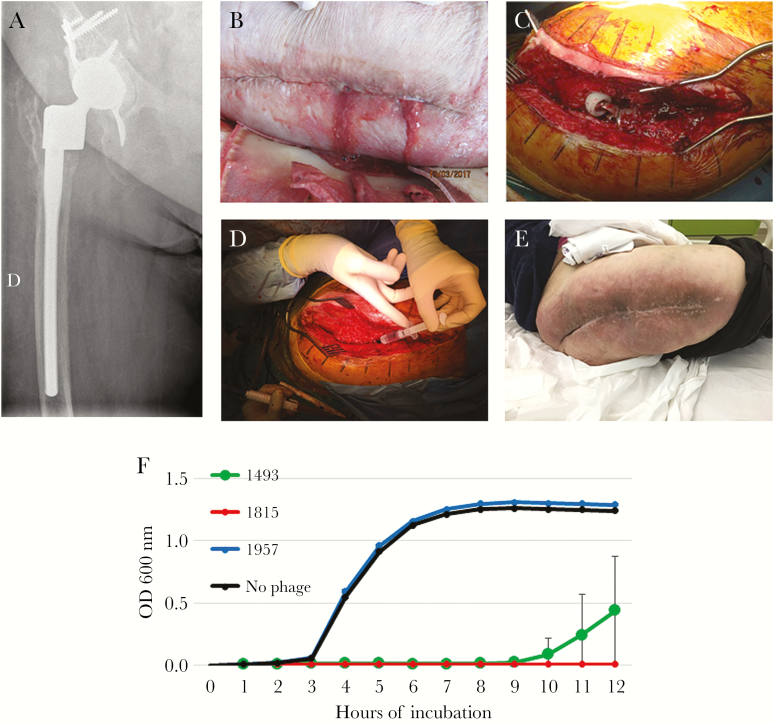
Massive resection prosthetic joint without prosthesis loosening (A) in a patient with purulent discharge (B) and relapsing *Staphylococcus aureus* right hip prosthetic joint infection. A debridement, antibiotics and implant retention (DAIR) procedure was performed (C), and a selected cocktail of *Pseudomonas aeruginosa* and *S aureus* bacteriophages was locally injected in the joint cavity at the end of the procedure (D). The outcome was favorable at 18 months (E). In the killing assay (F), the bacterial concentration over time of the strain without bacteriophages is indicated in black. The bacterial concentration over time of the patient’s strain in the presence of bacteriophages 1493, 1815, and 1957 at the highest multiplicity of infection is indicated in green, red, and blue, respectively.

## DISCUSSION

Prosthetic joint infection is the most dramatic complication of arthroplasty, leading to iterative surgeries, loss of function, considerable direct and indirect cost, and death. The treatment of staphylococcal chronic PJI requires prosthesis explantation to eradicate the biofilm, antibiotics, and then reimplantation in a 1- or a 2-stage strategy [1]. In elderly patients, explantation is sometimes not reasonable, especially in patients with large prostheses and with few motor disabilities. In such a population, suppressive antibiotic therapy is sometimes used after performing a “DAIR” procedure, but the rate of success at 2 years is only approximately 60% [2].

Bacteriophages are specific viruses that target bacteria [3]. They were first described in 1917 and remained a popular treatment throughout the 20th century in Eastern Europe, especially for patients with osteomyelitis [4]. By their nature, lytic bacteriophages are good candidates for antibacterial therapy. In comparison with antibiotics, they specifically target a bacterium, as long as it is present, and used it to amplify themselves. Indeed, the concentration of an antibiotic introduced into the human organism decreases rapidly with time (natural drug clearance from body), whereas phages continue to multiply, and then decreases after elimination of bacterial cells [3, 4]. This phenomenon, although observed in vitro and in nature, is unique and suggests that it could occur in humans. As a result, a single administration or a few administrations may theoretically be sufficient to treat a bacterial infection in humans. Bacteriophages remained a popular treatment in Eastern Europe (Georgia and Poland), especially for patients with osteomyelitis for whom traditional and preformed cocktails of bacteriophages are locally applied through the fistula [4]. Because their production in such countries currently does not follow the European GMP, bacteriophages are never used in patients with PJI, especially due to the risk of pyrogenicity. In Western Europe and the United States, medical health authorities consider that it is crucially important to respect GMP standards when producing bacteriophages for conducting clinical trials and targeting marketing authorizations and authorizing salvage therapy to guarantee the quality of the product.

In the European multicenter clinical trial, which was recently conducted by Pherecydes Pharma to evaluate phage therapy on burn wound infections, phages were produced according to GMP, but they are no longer available [5]. New GMP productions were not initiated yet. Therefore, GMP bacteriophages were not available. For this case, anti-*P aeruginosa* and anti-*S aureus* phages selected among the library of Pherecydes Pharma were produced in the R&D laboratory of the company. The major difference in the production process was not technical but related to the quality assurance level of the laboratory, which did not reach that of a GMP unit. This uncommon situation was accepted in this case of unmet medical need, but it implied a thorough evaluation of the quality control certificates of analysis of each bacteriophage by both ANSM and medical staff. They specifically evaluated the elimination of bacterial components (toxins etc) generated during the production process.

*Pseudomonas aeruginosa* was not retrieved in surgical samples, and the effect of the corresponding bacteriophages was difficult to evaluate. One of the 3 *S aureus* bacteriophages lacked efficacy on the patient’s strain, but the other 2 proved to be active. These findings show that it is desirable to isolate the strain infecting a patient before surgery (ie, by performing preoperative joint fluid culture) to perform a phagogram for selecting the active bacteriophage(s) before local injection. The use of bacteriophage is particularly promising in patients with PJI because bacteriophages and antibiotics are synergistic [6, 7], because some in vitro and animal models demonstrated that bacteriophages could have an anti-biofilm activity [6, 7], and because the rate of success, regardless of the clinical presentation (ie, acute or chronic), is unacceptably low [2, 8–12]. Finally, this salvage treatment was safe. The treatment success may have been due to the action of bacteriophages on the *S aureus* biofilm, because the patient had not received further antibiotics active against that organism for 12 months.

There is a considerable opportunity to develop the use of bacteriophages in patients with PJI in France because of the following: (1) it is now possible to select a bacteriophage mix through a susceptibility test (phagogram); (2) their production with a high level of purity according to European GMP is achievable; (3) ANSM agrees for the use of bacteriophages as salvage therapy; (4) our infectiologists and orthopedic surgeons from a reference center are motivated to recruit a large cohort of patients, including more complex cases that require salvage therapy; (5) our pharmacists agree to take responsibility to assemble a magistral preparation (mix of bacteriophages) just before the peroperative administration.

As a first step, it seems reasonable to limit this treatment in specialized units to patients (1) with PJI at high risk of complication in case of explantation and (2) for whom suppressive oral antimicrobial therapy is discussed. In addition to conventional therapies such as DAIR and antibiotics, the use of bacteriophages that may have an anti-biofilm activity, as suspected in the case reported here, may contribute to improvement of patients at particularly high risk for complication, long-term antibiotic toxicity, and mortality. It would be of great interest to assess the value of this treatment for patients with acute PJI. Finally, bacteriophages active on *Enterobacteriaceae* and coagulase-negative staphylococci (such as *Staphylococcus epidermidis*) produced according to GMP has to be considered, because these pathogens are frequently involved in patients with PJI and are more and more resistant to conventional antibiotics.

## CONCLUSIONS

The salvage use of a bacteriophage mix was safe and associated with a clinical success and a potential anti-biofilm activity in a patient with relapsing *S aureus* PJI. Selecting the best bacteriophage mix based on a phagogram of the infecting strain should be performed before bacteriophage therapy. Production of bacteriophages with a high purity level along GMP guidelines is currently possible, making the scientific evaluation of their potential clinical benefit in BJI feasible.

## Supplementary Data

Supplementary materials are available at *Open Forum Infectious Diseases* online. Consisting of data provided by the authors to benefit the reader, the posted materials are not copyedited and are the sole responsibility of the authors, so questions or comments should be addressed to the corresponding author.

Supplementary_Video_File

Supplementary_Video_Legend

## References

[1] Osmon DR, Berbari EF, Berendt AR, et al Diagnosis and management of prosthetic joint infection: clinical practice guidelines by the Infectious Diseases Society of America. Clin Infect Dis 2013; 56:e1–25. 23223583 10.1093/cid/cis803

[2] Prendki V, Ferry T, Sergent P, et al Prolonged suppressive antibiotic therapy for prosthetic joint infection in the elderly: a national multicentre cohort study. Eur J Clin Microbiol Infect Dis 2017; 36:1577–85. 28378243 10.1007/s10096-017-2971-2

[3] Clokie MR, Millard AD, Letarov AV, Heaphy S Phages in nature. Bacteriophage 2011; 1:31–45. 21687533 10.4161/bact.1.1.14942 PMC3109452

[4] Kutateladze M, Adamia R Bacteriophages as potential new therapeutics to replace or supplement antibiotics. Trends Biotechnol 2010; 28:591–5. 20810181 10.1016/j.tibtech.2010.08.001

[5] Jault P, Leclerc T, Jennes S, et al Efficacy and tolerability of a cocktail of bacteriophages to treat burn wounds infected by *Pseudomonas aeruginosa* (PhagoBurn): a randomised, controlled, double-blind phase 1/2 trial. Lancet Infect Dis 2018; pii: S1473-3099(18)30482-1. 10.1016/S1473-3099(18)30482-1 30292481

[6] Oechslin F, Piccardi P, Mancini S, et al Synergistic interaction between phage therapy and antibiotics clears *Pseudomonas aeruginosa* infection in endocarditis and reduces virulence. J Infect Dis 2017; 215:703–12. 28007922 10.1093/infdis/jiw632 PMC5388299

[7] Kumaran D, Taha M, Yi Q, et al Does treatment order matter? Investigating the ability of bacteriophage to augment antibiotic activity against *Staphylococcus aureus* biofilms. Front Microbiol 2018; 9:127. 29459853 10.3389/fmicb.2018.00127 PMC5807357

[8] Bouaziz A, Uçkay I, Lustig S, et al Non-compliance with IDSA guidelines for patients presenting with methicillin-susceptible *Staphylococcus aureus* prosthetic joint infection is a risk factor for treatment failure. Med Mal Infect 2018; 48:207–11. 29122410 10.1016/j.medmal.2017.09.016

[9] Lora-Tamayo J, Senneville É, Ribera A, et al The not-so-good prognosis of streptococcal periprosthetic joint infection managed by implant retention: the results of a large multicenter study. Clin Infect Dis 2017; 64:1742–52. 28369296 10.1093/cid/cix227

[10] Rodríguez-Pardo D, Pigrau C, Lora-Tamayo J, et al Gram-negative prosthetic joint infection: outcome of a debridement, antibiotics and implant retention approach. A large multicentre study. Clin Microbiol Infect 2014; 20:O911–9. 24766536 10.1111/1469-0691.12649

[11] Lora-Tamayo J, Murillo O, Iribarren JA, et al A large multicenter study of methicillin-susceptible and methicillin-resistant *Staphylococcus aureus* prosthetic joint infections managed with implant retention. Clin Infect Dis 2013; 56:182–94. 22942204 10.1093/cid/cis746

[12] Bouaziz A, Uçkay I, Lustig S, et al Microbiological markers suggesting high inoculum size at time of surgery are risk factors for relapse in patients with *Staphylococcus aureus* prosthetic joint infection. J Infect 2012; 65:582–4. 22982013 10.1016/j.jinf.2012.09.006

