# Supplementary material for: Salvage Debridement, Antibiotics and Implant Retention (“DAIR”) With Local Injection of a Selected Cocktail of Bacteriophages: Is It an Option for an Elderly Patient With Relapsing Staphylococcus aureus Prosthetic-Joint Infection?
Source: Open Forum Infect Dis. 2018 Oct 24;5(11):ofy269. doi: 10.1093/ofid/ofy269 (PMC6240628; doi:10.1093/ofid/ofy269)
Supplement: Supplementary_Video_Legend [file ofy269_suppl_supplementary_video_legend.docx]

**Supplementary file.** Video of the peroperative injection of the bacteriophage cocktail just after the DAIR procedure, just before joint closing.
